# A slow rainy season onset is a reliable harbinger of drought in most food insecure regions in Sub-Saharan Africa

**DOI:** 10.1371/journal.pone.0242883

**Published:** 2021-01-20

**Authors:** Shraddhanand Shukla, Greg Husak, William Turner, Frank Davenport, Chris Funk, Laura Harrison, Natasha Krell

**Affiliations:** 1 Climate Hazards Center, Department of Geography, University of California, Santa Barbara, California, United States of America; 2 Department of Geography, University of California, Santa Barbara, California, United States of America; Potsdam Institute for Climate Impact Research, GERMANY

## Abstract

Since 2015, Sub-Saharan Africa (SSA) has experienced an unprecedented rise in acute food insecurity (AFI), and current projections for the year 2020 indicate that more than 100 million Africans are estimated to receive emergency food assistance. Climate-driven drought is one of the main contributing factors to AFI, and timely and appropriate actions can be taken to mitigate impacts of AFI on lives and livelihoods through early warning systems. To support this goal, we use observations of peak Normalized Difference Vegetation Index (NDVI) as an indicator of seasonal drought conditions following a rainy season to show that delays in the onset of the rainy season (onset date) can be an effective early indicator of seasonal drought conditions. The core of this study is an evaluation of the relationship of the onset dates and peak NDVI, stratified by AFI risks, calculated using AFI reports by the United States Agency of International Development (USAID)-funded Famine Early Warning Systems Network (FEWS NET). Several parts of SSA, mostly located in East Africa (EA), reported the “Crisis” phase of AFI—requiring emergency food assistance—at least one-third of the time between April 2011 to present. The results show that the onset date can effectively explain much of the interannual variability in peak NDVI in the regions with the highest AFI risk level, particularly in EA where the median of correlation (across all the Administrative Unit 2) varies between -0.42 to -0.68. In general, an onset date delay of at least 1 dekad (10 days) increases the likelihood of seasonal drought conditions. In the regions with highest risks of AFI, an onset delay of just 1 dekad doubles the chance of the standardized anomaly of peak NDVI being below -1, making a -1 anomaly the most probable outcome. In those regions, a 2-dekads delay in the onset date is associated with a very high probability (50%) of seasonal drought conditions (-1 standardized anomaly of NDVI). Finally, a multivariate regression analysis between standardized anomaly and onset date anomaly further substantiates the negative impacts of delay in onset date on NDVI anomaly. This relationship is statistically significant over the SSA as a whole, particularly in the EA region. These results imply that the onset date can be used as an additional critical tool to provide alerts of seasonal drought development in the most food-insecure regions of SSA. Early warning systems using onset date as a tool can help trigger effective mid-season responses to save human lives, livestock, and livelihoods, and, therefore, mitigate the adverse impacts of drought hazards.

## 1 Introduction

Food insecurity is rapidly increasing, especially in Sub-Saharan Africa (SSA) [1, 2]. At present, according to the United States Agency of International Development (USAID)-funded Famine Early Warning Systems Network (FEWS NET), up to 113 million people in 46 countries, most of which are in SSA, are likely to require emergency food assistance in 2020, a projected 140% increase over 2015 (25% of which is attributed to COVID-19 pandemic-related impacts) [2]. Climate extremes such as droughts, along with conflicts and economic shocks, are the primary contributors to the record increase in acute food insecurity (AFI) in the last few years [2, 3]. For example, according to FEWS NET, droughts within SSA in 2015, 2016, and 2017 have led to substantial rises in populations needing emergency food assistance [4–8]. These increases underscore an ongoing need for improved drought early warning to support early warning of AFI needed to mitigate the adverse impacts of AFI on vulnerable lives and livelihoods. Here, we demonstrate that the onset of the rainy season (referred to in this study as onset date) offers a unique drought early warning opportunity. The onset date can be tracked using satellite and in situ-based rainfall observations [9] and provides the first observational glimpse into a crop growing season and how the season may evolve. It can also be forecasted using weather (up to 2 weeks in advance) or subseasonal scale (up to 4 weeks in advance).

Early identification and warning of drought conditions can trigger well-targeted and crucial relief intervention to mitigate socio-economic losses and save lives at risk of AFI [10]. Furthermore, advance notice of developing food insecurity conditions allows for the pre-positioning of relief, followed by prudent and timely distribution of aid, thus preventing the catastrophic disruptions of food and labor markets that can lead to widespread famine. For example, according to a report from Food and Agriculture Organization’s Food Security and Nutrition Analysis Unit report and partner agencies, during 2010–2011, when the capacity to act based on early warning was limited, a severe drought in Somalia led to widespread famine [11] and resulted in 244,000 to 273,000 lives lost. In contrast, a drought of similar severity in Somalia in 2016–17 [12, 13] resulted in limited increases in mortality, thanks to large-scale intervention triggered by early warning of the drought event. As the 2017 rains failed, increased food aid for millions was arriving in Somalia, preventing a catastrophic rise in food prices, as was experienced in 2011 [12]. And yet, more effective, proactive, and earlier-early warning is still needed. For example, between October and December of 2016, a severe drought led to massive livestock losses [14, 15] for millions of East African pastoral households—very vulnerable populations dependent on cattle, sheep, camels, and goats as their main source of livelihood. These herds represent a financial reserve, as well as an immediate source of food and income. Their destruction reinforces a cycle of poverty. Improved predictions of drought conditions that lead to poor pastoral and crop conditions can support more effective mitigation. Lagged relationships between early-season rainfall conditions and late-season vegetation health provide one valuable approach to the prediction of drought conditions.

An optimal early warning system relies on a gradient of increasing alarm issued at different times in the season [10]. Given the role of climate extremes, such as droughts, in the development and progression of food insecurity [16], substantial focus is given to monitoring and forecasting droughts using rainfall [9], vegetation [17–19], and hydrologic conditions [20–24]. Several months before the season, early warning of food insecurity considers large-scale climate (e.g., sea surface temperatures, precipitation, and temperature) forecasts [25, 26]. Although useful for providing the earliest warning of food insecurity, climate forecasts typically suffer from limited skill and inherent uncertainty [25, 27, 28]. During the season, the early warning is based on accurate and routine monitoring and forecasting using several different satellite, model, and station-based data sets [9, 21, 29–31] along with weather [32] and subseasonal [33] to seasonal forecasts [8, 20, 24, 25]. These data sets and tools used during the season can help provide spatially detailed and accurate drought assessments but are hampered by limited lead times, as it is only near the end of the rainy season that satellite vegetation or rainfall data may be capable of accurately identifying drought conditions and poor harvests. As the season approaches its end, early warning derived from observation-based monitoring of the overall season and field reports, including existing livelihood, market, and nutrition conditions, allows for a skillful assessment of food insecurity.

How the crop growing season evolves has an impact on labor demand and supply during the season and the availability of food thereafter, and so, it has an influence on food-insecurity conditions during and after the season. The onset date is also critical for various cropping-related decisions, such as purchasing seeds and fertilizers, choosing planting time, etc. The onset date provides an opportunity for early warning of food insecurity, which falls several months in advance of the harvest period and the next lean season. Therefore, in this study, we investigate the relationship between the onset date and drought conditions following the rainy season in SSA. For the purpose of this study, we focus on the second sub-national Administrative unit (AU2) in those countries of SSA that are currently being monitored by FEWS NET and are historically prone to AFI (for Kenya, we are using AU2 as per 2009 classification). The spatial scale of AU2 is chosen for this analysis to make the results of the study directly relevant for decision-making, as crop yield reports and food insecurity assessments are typically made at this scale. Furthermore, we consider anomalies of peak NDVI following a rainy season to be the indicator of seasonal vegetation drought conditions. Vegetation drought conditions at this time can imply poor agricultural harvest, as well as rangeland conditions (for pastoral usage), both of which contribute to food insecurity.

Several past studies have focused on either the onset date or the start-of-season (SOS), which is typically estimated using vegetation phenology based on remotely sensed vegetation data sets. These past studies have analyzed the onset date [34–39] or focused on predicting the onset date for different regions in SSA [40–42]. Likewise, several past studies have examined changes in SOS and other characteristics of vegetation phenology [43–50]. However, thus far, to our knowledge, there has not been any investigation of a possible linkage between the onset date and seasonal drought conditions in food-insecure SSA areas to support AFI early warning.

## 2 Methods

Here, we describe our methodology for calculating levels of AFI risks in SSA, for identifying onset date, and for estimating peak NDVI, which is used as an indicator of seasonal drought conditions for the purposes of this study.

### 2.1 Classifying regions based on risks of acute food insecurity

To identify the regions in SSA at the highest risk of AFI, we rely on FEWS NET’s archive of quarterly reports of the current situation of AFI between April 2011 and February 2020. FEWS NET uses the Integrated Food Security Phase Classification (IPC) to identify AFI. The IPC, a multi-partner initiative that currently includes 15 organizations, provides evidence- and consensus-based classification of food insecurity and acute malnutrition conditions to support effective decision-making for emergency responses, as well as long-term management decisions. The IPC provides AFI classification to support short-term emergency responses, and to prevent, mitigate, or decrease food insecurity that threatens lives and livelihood. As per IPC, AFI varies from phase 1 to 5, where phase 1 indicates “Minimal” phase, 2 indicates “Stressed,” 3 indicates “Crisis,” 4 indicates “Emergency,” and 5 indicates “Famine” phase of food insecurity.

In this study, we focus on the regions that frequently reported IPC phase 3 “Crisis” or worse from April 2011 to February 2020. This threshold was selected because urgent humanitarian assistance is needed when AFI reaches the “Crisis” or worse phase. The IPC describes the “Crisis” phase as meaning households either “Have food consumption gaps that are reflected by high or above-usual acute malnutrition; or Are marginally able to meet minimum food needs but only by depleting essential livelihood assets or through crisis-coping strategies” [51]. Household hunger at this phase is considered to be “moderate,” with the severity of hunger worsening with the higher phases.

For the purpose of this study, AU2 in SSA are classified in terms of risks of AFI, based on the frequency (in percentage of time) with which a given AU2 reported “Crisis” or worse IPC phase during April 2011 and February 2020. Table 1 lists the different levels of AFI risks and associated frequency of the reports of “Crisis” or worse IPC phase. Here, AU2 with AFI risk level 5 are considered to be the most food insecure, and AU2 with AFI risk level 1 are considered to be the least food insecure.

**Table 1:** The scheme for classifying AU2 in Sub-Saharan Africa in to different levels of Acute Food Insecurity (AFI) risks.

AFI risk category	Frequency of “Crisis” or worse IPC phase reports from April 2011 to February 2020
AFI level 1	<1%
AFI level 2	1 to 10%
AFI level 3	10 to 20%
AFI level 4	20 to 33%
AFI level 5	>33%

### 2.2 Identifying onset of the rainy season (onset date)

Numerous definitions exist for identifying the start of the season. In past studies, the start of the season is either considered as the start of the rainy season, and hence is identified based on rainfall data [34, 36, 40–42], or it is identified based on vegetation phenology, often using satellite-based vegetation condition estimates such as NDVI [39, 44, 45, 48, 52].

For the purposes of this study, to identify the onset date, we use a simple heuristic designed to coincide with the planting by farmers. This scheme is employed at a grid cell scale (0.1 deg latitude x 0.1 deg longitude) and uses the Climate Hazards Center InfraRed Precipitation with Stations (CHIRPS) precipitation data set [9]. To identify the onset date for a given grid cell, this scheme starts its search for the onset three dekads before a traditional onset of a rainy season. It searches for the first dekad with at least 25 mm of rainfall, followed by two dekads, which sum to 20 mm of rain. If both thresholds are met, the first dekad of that three-dekad series would be considered the onset date. If either threshold is unmet, a search for the onset date continues. This scheme for identifying onset date, originally proposed by AGRHYMET, is widely used in the region, as it is employed by the Water Requirement Satisfaction Index (WRSI) model [31]. The gridded implementation of the WRSI model was developed to provide estimates of crop conditions to support food-insecurity early warning. Gridded implementation of this model allows for input from satellite-based data sets (such as rainfall and evaporative demand) which, in SSA, are often more routinely and readily available than in situ reports. The WRSI model has been in operational application for supporting food insecurity early warning for nearly two decades [31]. The United States Geological Survey’s Earth Resources Observation and Science (USGS EROS) center provides regular updates of expected crop conditions in SSA, based on this model.

Following the conventions used in the WRSI simulations for routine operational monitoring, this study focuses on regions of East Africa (EA), West Africa (WA), and Southern Africa (SA) that are included in the operational monitoring, and more particularly, on the main rainy seasons in those regions. For EA regions, this study considers two seasons: “long rains,” which includes the region that experienced rains during the March-May, as well as the June-September season, and “short rains,” which includes the regions that experienced another rainy season during the October-December season. S1 Fig shows the regions and seasons included in this analysis, as well as climatological onset month (left panel) for each region and season.

### 2.3 Identifying peak NDVI following a rainy season

The NDVI has been widely used to monitor drought conditions [52], estimate changes in land management [53] and vegetation and crop conditions [52, 54], and estimate crop yield [46]. During a growing season, the NDVI (especially in the case of crops and other non-perennial vegetation) typically reaches its peak value following the main rainfall accumulation period of the season [45]. The FEWS NET employs eMODIS NDVI [30] for the routine monitoring of drought conditions to support food-insecurity early warning. The USGS EROS provides regular updates and access to gridded eMODIS data for drought monitoring purposes. NDVI is also one of the main inputs to GEOGLAM’s Crop Monitor for Early Warning (CM4EW) that provides crop classification maps for food-insecure regions, as well as major crop-producing regions across the globe [55, 56]. Given the widespread use of NDVI as an indicator of agricultural drought, we believe that the choice of NDVI for this study is appropriate, as it helps make the results of this study directly relevant to early warning communities that rely on NDVI as one of the main indicators. However, we do acknowledge that for certain special cases, such as the cases where dry biomass is used for livestock, NDVI is likely not a suitable indicator. In this study, we use “end-of-season” maximum NDVI (referred to here as “peak NDVI”) as an indicator of drought conditions most relevant to food-insecurity conditions following the season. The primary reasons for selecting maximum NDVI at the month of climatological maximum are (a) climatological maximum NDVI captures the time of the season, after the peak of the rainy season, when vegetation has had a chance to respond to the timing and amount of seasonal rain, (b) providing a temporal window (of 3 dekads) to select the peak, allowing for variability in the timing of vegetation response, and (c) it avoids a false peak early in the season responding to some early surge in rainfall.

**Fig 1:**
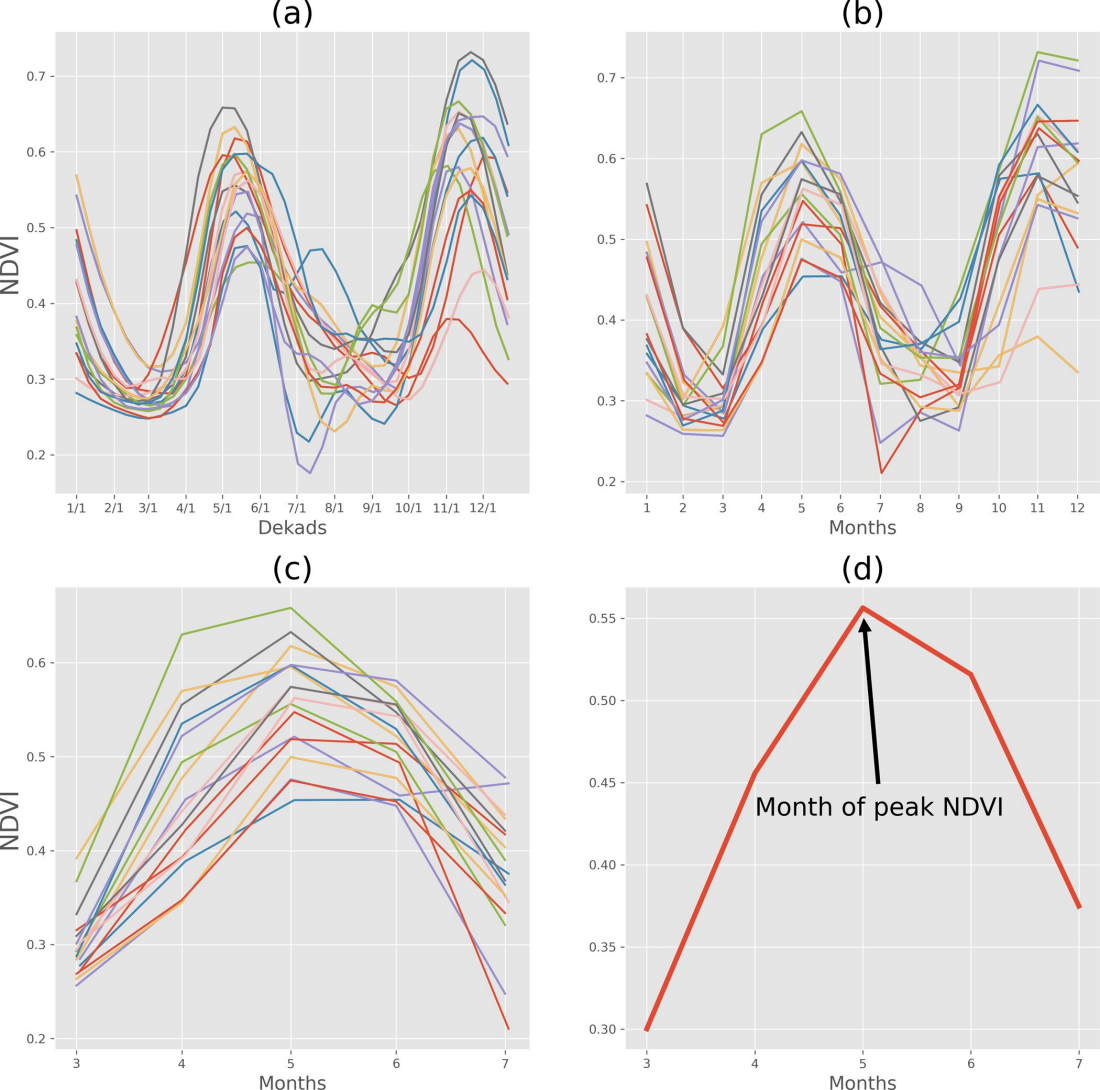
Approach for identifying month with peak NDVI demonstrated using an AU2 in Somalia as an example. (a) Dekdal NDVI between 2003–2018 (b) monthly NDVI (i.e., max dekadal NDVI in a given month) and (c) monthly NDVI for the months within the prescribed 4-month seasonal window from the climatological onset month of March to the end of season month of July (d) climatological mean monthly NDVI for months in (c). Month of peak NDVI is the month that has the highest NDVI during the prescribed seasonal window starting on the climatological onset of the rainy season.

Fig 1 demonstrates an example of how climatological maximum NDVI month is identified and peak NDVI for a given season is calculated. The steps involved in identifying peak NDVI during each year are:

1. Extract dekadal eMODIS NDVI spatially aggregated over any given AU2 from USGS’s Early Warning Explorer. Fig 1A shows the dekadal NDVI for 2003–2019 for an AU2 in Somalia.
2. Convert dekadal NDVI to monthly NDVI using the “maximum” of all dekadal NDVI values in a given month. Fig 1B shows the monthly NDVI for 2003–2019.
3. Calculate the month of “climatological” onset date by calculating the median of the “onset date” values for all years in 2003–2019, the period of record for the eMODIS data set. S1 Fig (left panel) shows the climatological onset month (month of onset date) for each of the regions and seasons considered in this study.
4. Calculate the month of “climatological” end of the season (EOS) by adding a predefined seasonal window to the month of “climatological” onset month. The predefined window was 4 months for “long rains” and “Short rains” seasons in EA, and 7 months for other seasons and regions. A predefined window is assigned to limit the search for “maximum” NDVI from a month to months within and just following the rainy season. This step assists with the separation of bimodal rainfall seasons and associated twice-a-year peak NDVI, as it is in the case of the AU2 NDVI for Somalia presented here.
5. Extract monthly NDVI values for the period between the “climatological” month of onset and EOS for all years. Fig 1C shows the monthly NDVI between the climatological onset month of March and EOS month of July (4 months after the “climatological” onset month).
6. Convert the monthly mean NDVI values between the “climatological” onset month and EOS months over the 2003–2019 period to get “climatological” mean value of monthly NDVI for each month, as shown in Fig 1D. Finally, using this time series of “climatological” monthly mean NDVI values, identify the month with highest mean monthly NDVI. We assign that month to be the month of peak NDVI. S1 Fig (left panel) shows the climatological onset month for each of the regions and seasons considered in this study. S1 Fig (right panel) shows the month of peak NDVI following each of the rainy seasons considered in this study.
7. Extract the monthly NDVI values (as in Fig 1D) for the month of peak NDVI from all years of 2003–2019.

### 2.4 Regression between standardized NDVI anomaly and onset date anomaly

We also fit a series of regression models with the goal of identifying the degree with which one or more dekad delay in onset date can influence NDVI standardized anomalies (NDVI^Z^). Specifically, we fit two sets of models. In the first model (eq. [1]), we regress NDVI^Z^ on onset date anomalies (based on median onset dekad), and in the second model (eq. [2]), we regress NDVI^Z^ on an indicator variable set to one if the onset date is greater than two dekads late, and zero otherwise. The first model allows us to estimate the impact of a one-dekad change in onset date on NDVI^Z^, while the second model allows us to estimate the specific impact of onset date being greater than two dekads late. Both models include a time trend (year), and dummy variables (fixed effects) for country, and region/season (i.e., separate dummies for East African long and short seasons). The time trend adjusts for unobserved factors varying across time, while fixed effects for region/season and country control for unobserved factors that vary within regions and countries.

Formally the models are:

$${NDVI}_{\left(i,t\right)}^{Z}=\beta_{0}+{Years}_{\left(t\right)}+Regian/Season_{\left(r\right)}+Country_{\left(c\right)}+\beta_{1}Onset\text{​}anomaly_{\left(i,t\right)}+\in_{\left(i,t\right)}$$ (1)

$$NDVI_{\left(i,t\right)}^{Z}=\beta_{0}+Years_{\left(t\right)}+Regian/Season_{\left(r\right)}+Country_{\left(c\right)}+\beta_{2}OnsetEarly/Late_{\left(i,t\right)}+\in_{\left(i,t\right)}$$ (2)

where i indexes administrative units, t indexes time (years), *β*_0_ is a constant and the main coefficients of interest are *β*_1_ (for the regression on continuous Onset date anomalies) and *β*_2_ (for the regression the Onset early/late indicator), *Region/Season* [*Country*] is a dummy variable for region/season and country control.

For each equation, we run a model collectively on all the regions/seasons combined (See, S1 Fig), as well as separate models for each individual region and season. Note that the region dummy variable “*Region/Season*” is removed in the region-specific models. We adjust standard errors (*∊*) for spatial and serial correlation by using a two-way clustering approach [57]. Specifically, we cluster on country and year, allowing for arbitrary spatial correlation and heteroskedasticity within a given year (across all observations in that year), and for arbitrary serial correlation and heteroskedasticity for all admin units within a given country. This follows the “state-year” approach outlined in [57]. In addition, we make degrees of freedom corrections (based on the number of clusters) similar to those defined in [58] and evaluated for a dynamic spatial panel setting, as in [59].

Finally, in order to explore the spatial variation in estimated coefficients, we run a separate time series model for each administrative unit, similar to eq. [1] but without the country or region fixed effects (We cannot run eq. [2] for each individual administrative unit because not every administrative unit has enough variation (‘late’ seasons) to construct the indicator). In this case, we adjust the standard errors for heteroskedasticity and serial correlation (HAC) [60]. We plot the estimates (*β*_1_) for each administrative unit time series to model and provide some context for how much the response of NDVI^Z^ to Onset date anomaly varies across the space.

## 3 Results

We first highlight the levels of AFI risks in different regions of SSA, which are used to identify the value of the onset date as a drought early warning tool, especially over the most food-insecure regions of SSA. Following this, we investigate the covariability of onset dates and peak NDVI, shifts in NDVI at extreme ends of onset dates, and variation in distributions of peak NDVI at different ranges of delayed and early onset dates to establish the value of onset dates as an indicator of drought conditions.

### 3.1 State of acute food insecurity in Sub-Saharan Africa

FEWS NET uses the IPC AFI phase classification system to provide routine reports (at quarterly frequency until 2016 and triannually thereafter) on current situations (and near- as well as medium-term outlook) of food insecurity in several of the regions around the globe that are prone to food insecurity. It covers 22 countries in Africa. The routine reports (available since April 2011, when the current IPC classification was implemented) provide nearly a decade-long record of food insecurity conditions in SSA. Based on the reports on “current situations'' of food insecurity, which is the best surrogate available to “observations,” we identify (i) regions in SSA that have most frequently reported “Crisis” phase of food insecurity, and hence, are most food insecure, and (ii) the changes in this phase of food insecurity.

**Fig 2:**
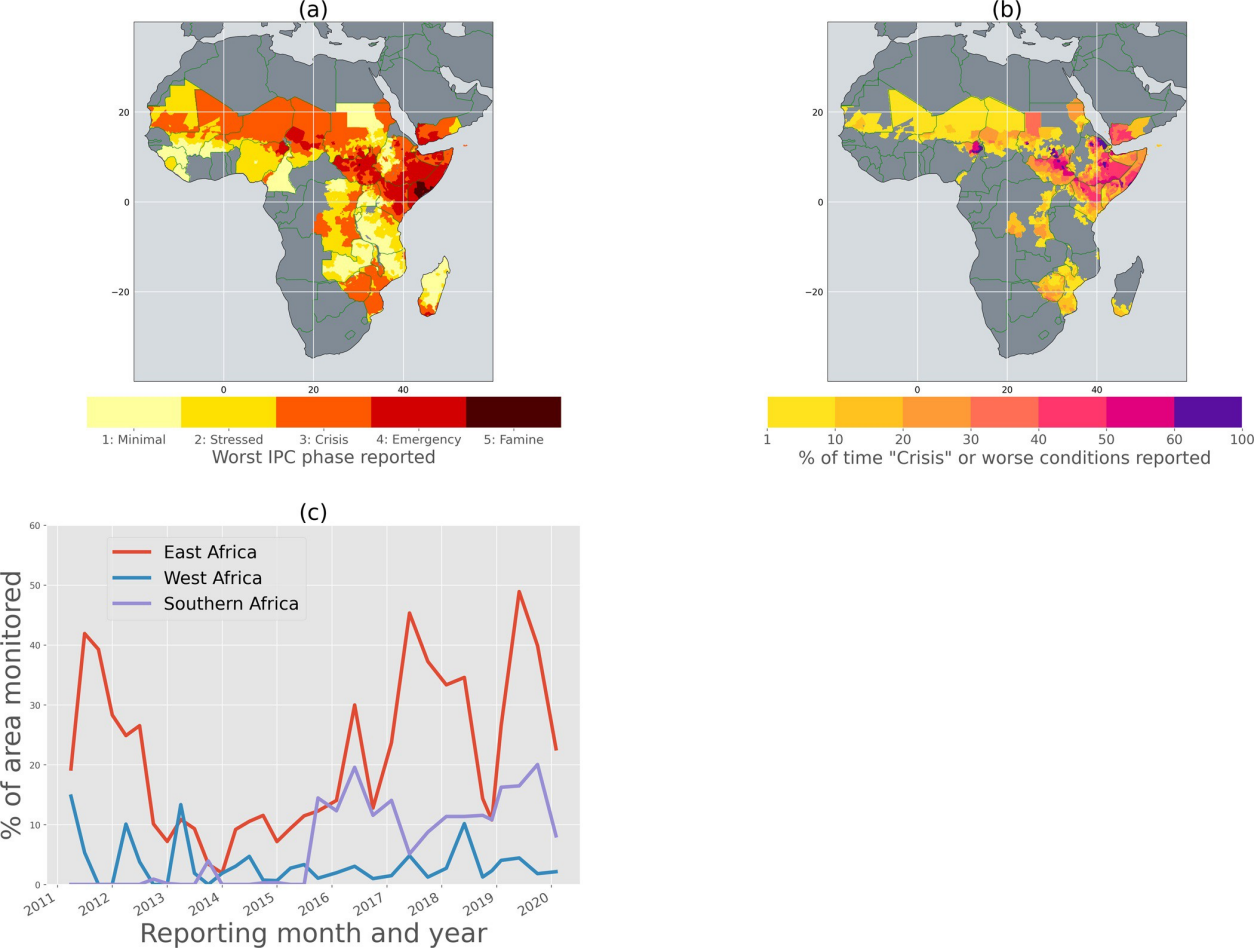
(a) Worst Integrated Phase Classification (IPC) acute food insecurity phase reported between April 2011 to February 2020 (b) Percentage of time during April 2011 to February 2020 that a given region reported “Crisis” (IPC phase 3) or worse phase of acute food insecurity (c) Percentage of FEWS NET-monitored areas in East, West, and Southern Africa that reported “Crisis” (IPC phase 3) or worse phase of acute food insecurity during this period.

Fig 2A shows the worst category of IPC phase reported in different parts of SSA since April 2011. Most of the countries monitored by FEWS NET reported “Crisis” phase (IPC class 3) food insecurity at least once, especially countries in EA and WA. EA stands out as a region with a large portion of countries reporting the worst two phases of food insecurity. Most of this region reported the phase of “Emergency” (IPC class 4) and even “Famine” (IPC class 5) at least once during this period. Note that the drought in 2011 led to famine conditions resulting in the deaths of more than 260,000 Somalis [11].

Fig 2B shows the frequency (measured as percentage of reports between April 2011 and February 2020) with which each of the regions in SSA reported “Crisis” (IPC phase 3) or worse conditions. This figure emphasizes the susceptibility of EA to AFI. Since April 2011, the majority of Somalia, South Sudan, Sudan, and substantial parts of Ethiopia and Kenya have reported “Crisis” conditions at least 30% of the time.

Fig 2C shows the temporal progression of the percent of FEWS NET-monitored areas in EA, WA, and SA that reported “Crisis” or worse phases of food insecurity from April 2011 to February 2020. The percentage of area is calculated relative to the area that FEWS NET monitored in each of these regions at any given time step. The EA region’s higher susceptibility to food insecurity, relative to the other regions, is once again demonstrated in this figure, as the percentage of areas reporting “Crisis” or worse conditions in EA is always higher than in WA and SA. Since 2015, at least 10%, and in 2019, up to 50%, of the region reported “Crisis” or worse phases of food insecurity. Since the beginning of 2016, the SA region has seen a substantial increase in food insecurity reaching up to 20%, although the percentage of area reporting “Crisis” or worse food insecurity was relatively low for the first 4–5 years. These large increases in SA are mostly due to multiple drought events in the region, including the strong El Niño-driven 2015–16 drought, which was followed by several additional drought events in 2018 and 2019. This increase in areas reporting “Crisis” phase since 2015 has translated into unprecedented increases in the number of people in need of emergency food assistance [2, 15, 61].

### 3.2 How well can variability in onset date anticipate variability in peak NDVI?

**Fig 3:**
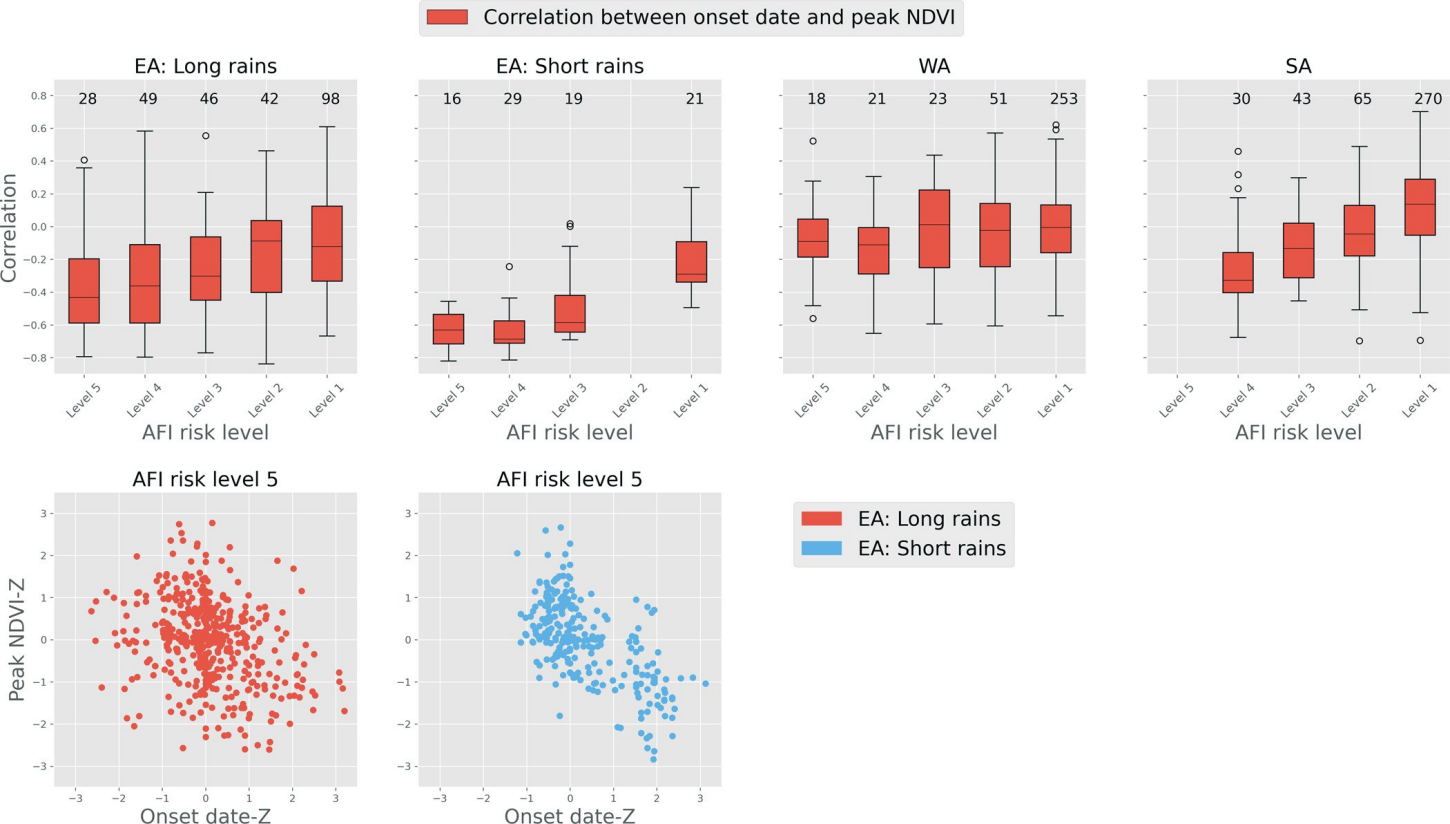
Top: Whisker plots describing the variability of correlation between timing of onset of the rainy season (onset date) and peak NDVI in the following months for East, West, and Southern Africa based on AFI risks (Table 1). Bottom: Scatter plots of standardized anomaly of onset date timing and peak NDVI for the AU2s with AFI risk level 5. *The number on the top of box-whisker plots indicate the number of AU2 in each of the corresponding bins. AFI risk level 1 indicates lowest risk of food insecurity and level 5 indicates highest risk, further explanation for AFI risk level can be found in Table 1.

Next, we examine the relationship between the variability of onset date and peak NDVI to assess how well onset date can provide early warning of drought conditions. Whisker plots in Fig 3 describe the variation in the correlations between onset date and peak NDVI for all focus regions and seasons. The correlation values are binned based on the level of AFI risk level (Table 1). Binning of correlation values based on level of AFI risk helps highlight the potential value of any relationship between onset date and NDVI for supporting early warning of food insecurity in the regions with the highest risks of food insecurity.

Fig 3 shows that generally, the correlation between onset date and NDVI tends to be strongest (negatively) for AU2 in AFI risk level 5 (most food insecure), which are mostly in the EA region. Of note is the fact that within the EA region, the correlation between onset date and NDVI is strongest during the OND (i.e., “short rains”) season. This is further highlighted by the scatter plots between onset date and peak NDVI for EA seasons in the most food-insecure regions (Bottom, Figs 3 and S2). These results (Bottom, Figs 3 and S2) show a clear (especially in the case of EA “short rains”) negative relationship between the onset date anomaly and NDVI anomaly. It is important to note that the difference in the strength of the relationship (as measured by correlation) between onset date anomaly and NDVI anomalies in EA during both seasons only indicates that the onset date may be a more reliable indicator of NDVI in “short rains” seasons (i.e., agricultural drought conditions), and that this result has no bearing on the AFI risks following each of the seasons.

Fig 3 also shows that, as the level of AFI risk decreases, in general, the median of correlation between onset date and NDVI gets weaker (closer to 0). This suggests that chronically food-insecure regions are also areas where onset of rainfall is strongly correlated with biophysical vegetation production. Nonetheless, even in the case of AU2 in AFI risk level 4, the median of correlation between onset date and NDVI generally stays negative across all of the regions between -0.1 to -0.6. In the case of AU2 with AFI risk level 1 (least food insecure), the median correlation is slightly above or close to 0 in SA and WA. The variability of the median correlation with the changes in the risks of food insecurity is most apparent in the case of EA, followed by the SA region. In other words, in the case of EA and SA, the median correlation is stronger negatively with AFI risk level 5, which becomes weaker as the risk of AFI decreases.

The strong negative correlation between onset dates and peak NDVI in the most food-insecure regions of SSA is at least partially related to the negative covariability between the onset dates and seasonal precipitation totals. The strong positive covariability between seasonal precipitation total and peak NDVI, as shown in S3 Fig, displays the link from onset date to rainfall anomaly and then NDVI. S3 Fig highlights the variability of correlation between onset dates and seasonal precipitation, as well as seasonal precipitation and peak NDVI, across regions with different rates of AFI risk. In the AFI risk level 5 regions of EA, the median correlation between onset dates and seasonal precipitation varies between -0.58 to -0.74, whereas the correlation between seasonal precipitation and peak NDVI varies between 0.7 to 0.74. The negative correlation between onset dates and seasonal precipitation is at least partially related to the short length of the rainy seasons, which makes the recovery in seasonal rainfall less likely in case of onset delay.

Additionally, in general the correlation between onset dates and seasonal precipitation and the correlation between seasonal precipitation and peak NDVI decreases as the AFI risk level decreases, indicating lower vulnerability to food insecurity in those regions.

### 3.3 How does NDVI vary at the extreme ends of onset date?

While the prior section focused on the correlation between the onset date and peak NDVI, here, we instead focus on how peak NDVI varies in the case of onset date extremes. We hypothesize that even in cases where the correlation between onset date and peak NDVI is weak, peak NDVI may be different when the onset date is at the extreme ends (i.e., earliest onset date vs. latest onset date).

**Fig 4:**
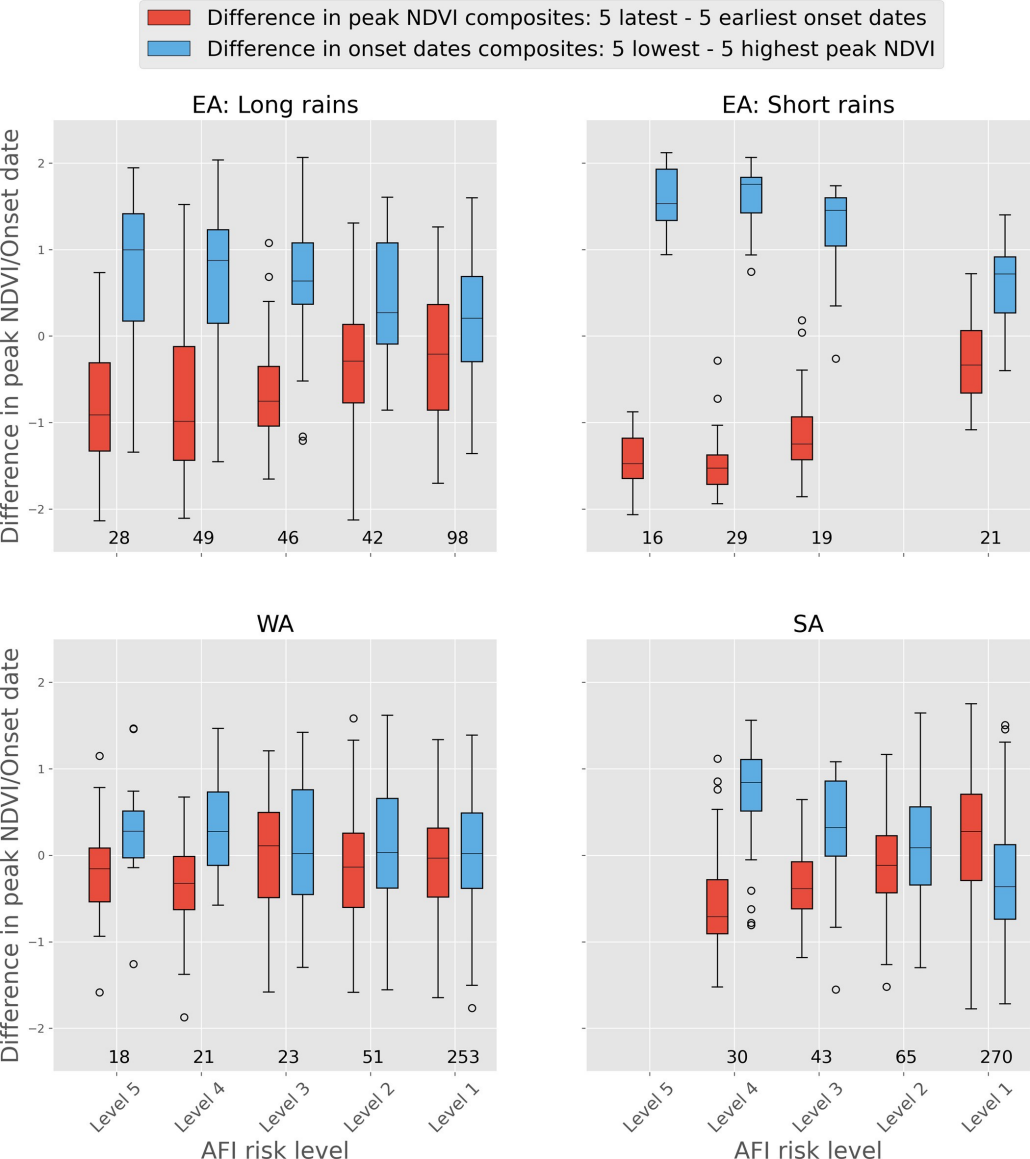
Difference in the mean of peak NDVI^Z^ composites based on earliest versus latest five onset date, and difference in the mean of onset date composites based on lowest versus highest five peak NDVI values, binned based on AFI risk level.

Fig 4 shows the variation in the differences of the mean of peak NDVI values during the 5 earliest and the 5 latest onset dates during the time period of 2003–2019 for all regions and seasons. Like the previous results, these differences have been binned based on the level of food insecurity risks. Additionally, in order to further test the relationship between onset date and peak NDVI, the differences in onset date based on peak NDVI composites are also presented. This is done by plotting the difference between the onset date values corresponding to the 5 lowest NDVI values and 5 highest NDVI values (i.e., peak NDVI extremes).

The influence of delay in onset date on peak NDVI is clear from these results as well, especially over the AU2 with AFI risk level 5 and level 4. These results show that NDVI tends to be smaller (red box plots) in the cases of 5 latest onset date than in the cases of 5 earliest onset date, which is highlighted by median NDVI values being below 0 in all cases and near or below -1 standard deviation in the case of the EA region.

This influence of onset date on NDVI is further substantiated by the differences in the onset date composites (light-blue box plots) corresponding to the 5 lowest and 5 highest NDVI. The median differences in onset date are almost always positive for the AU2 with AFI risk level 5. In the case of EA, this difference is typically between 1 to 2 standard deviation. Finally, the influence of onset date and NDVI, as per the results of this study, can be seen in the case of WA and even more strongly in the case of the SA region, especially for AU2 with AFI risk level 4 and 5.

### 3.4 How much delay in onset date is needed for drought?

**Fig 5:**
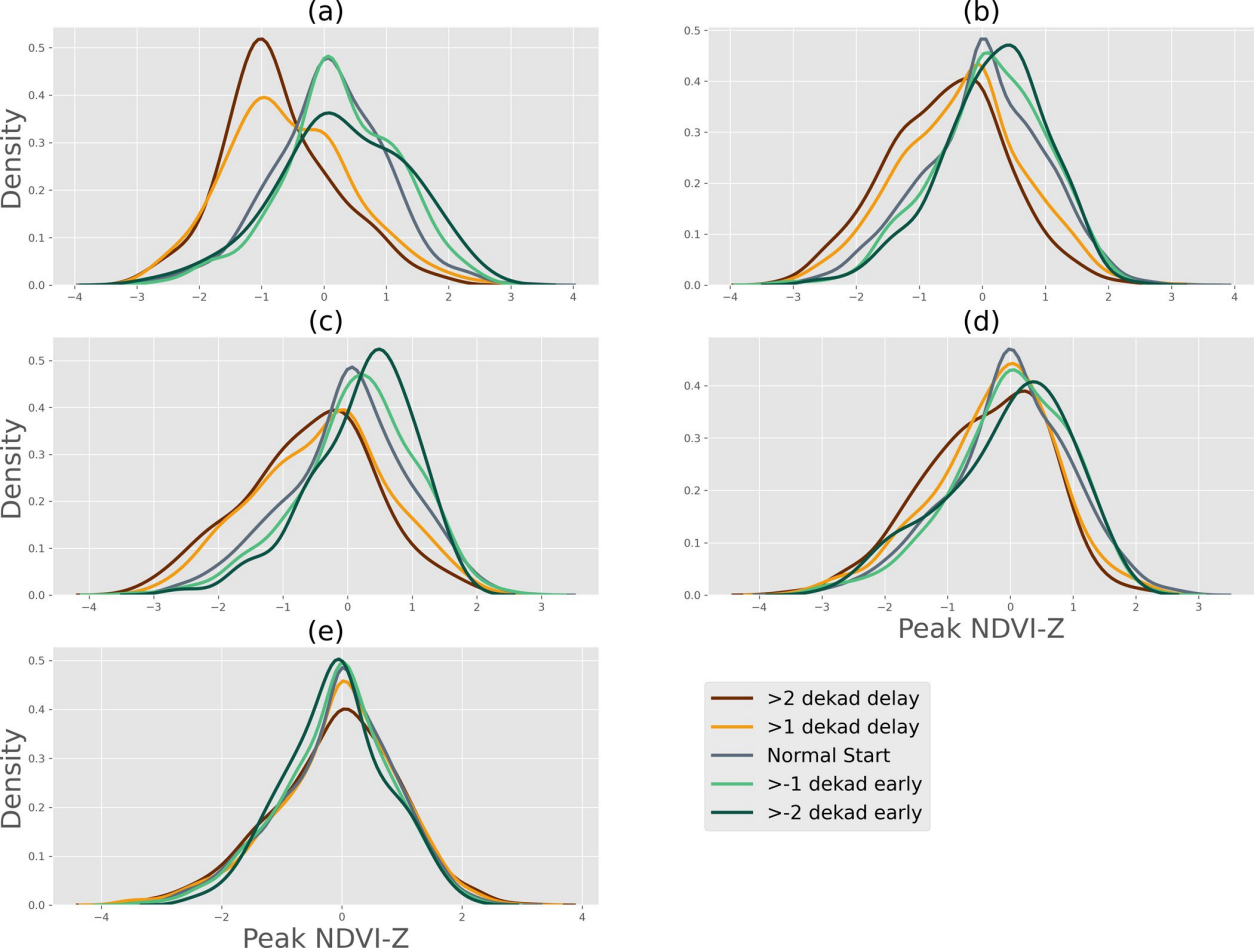
Difference in probability distributions of NDVI^Z^ when onset date was delayed versus normal versus early, for the AU2s in Sub-Saharan Africa with AFI risk (a) level 5, (b) level 4 (c) level 3, (d) level 2, (e) level 1.

**Fig 6:**
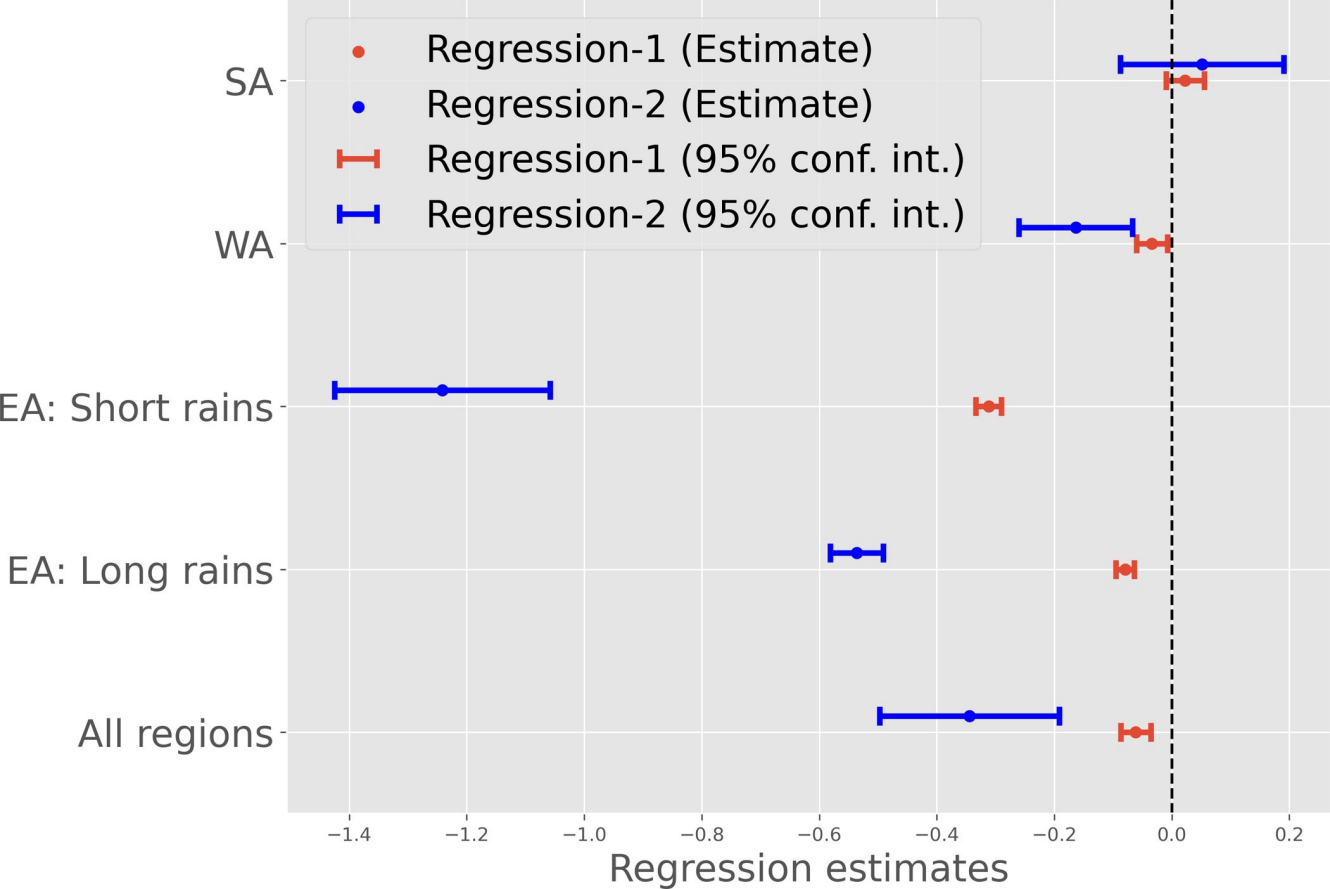
Regression estimates (and 95% confidence intervals) between standardized NDVI anomaly (NDVI^Z^) and (a) absolute onset date anomaly (“Regression-1”- see eq. [1]) and (b) discreet onset date anomaly (“Regression-2”- see eq. [2]), where onset anomaly is treated as a binary variable of value 0 (on time or early onset) or 1 (more than 2 dekad delay in onset date). We use two-way clustering to adjust confidence for both serial and spatial correlation; see section 2.4 for more details.

The results thus far have demonstrated the relationship in the variability of onset date and peak NDVI, and the influence of onset date extremes on peak NDVI, especially in the regions with the highest level of food insecurity risks. Here, we address a more practical question with direct implications for operational drought early warning: “How much delay in onset date is needed for drought development”? We address this question through two analyses (i) by comparing the distribution of NDVI^Z^ (calculated over 2003–2019 period) when the season is delayed (>1 or >2 dekad delay), versus when the season starts on time (between 1 dekad delay to 1 dekad early start), versus when the season starts early (>1 or >2 dekad early start) (Fig 5) and (ii) by performing a multivariate regression between onset date anomaly and NDVI^Z^ (Fig 6).

#### 3.4.1 Difference in probability distributions of standardized anomaly of peak NDVI based on onset date anomaly

For the first analysis, we grouped regions and seasons together and binned them based on the AFI risks (Table 1). This grouping is reasonable, as, in general, our results thus far indicate that AU2 with highest food-insecurity risks have comparable relationships in onset date and NDVI across different regions of SSA. It also allows us to increase the sample size of NDVI for a given onset date, which enables us to better estimate the distribution of NDVI^Z^.

Fig 5 shows the distribution of NDVI^Z^ in cases of delayed, normal, and early start of the season. These comparisons have been shown separately for AU2, with different levels of food insecurity. The results show that. especially in the case of AU2 with AFI risk level 5, there is a substantial shift in the distribution when the season is delayed by 1 or 2 dekads versus when it starts normally or early. When the season starts just one dekad late, the mode of the distribution shifts to -1 standardized anomaly of NDVI, whereas in the case of normal start, the mode is near 0, and in the case of early start, it is slightly above 0 (Fig 5A). The results also indicate that the chances of above-normal NDVI (area right of 0) are reduced in case of delayed start. For example, in the case of the AU2 with AFI level 5 (Fig 5A), the chances of NDVI being below -1 increase by around 200% if the onset date is delayed by more than 1 dekad.

The results for AU2 with AFI risk level 4 are similar, as the distribution of standardized anomaly shifts to the left of 0 indicates higher chances of below-normal NDVI relative to the cases with normal or early start; however, in this case, the uncertainty is greater. Finally, except for the case of AU2 with the AFI risk level 1, the differences in the probability distribution of NDVI^Z^ for “normal” onset is statistically different from the distribution in the case of delayed onset, as per two-sample Kolmogorov–Smirnov test (p<0.025). In general, KS test statistic (D) decreases as AFI risk level decreases, with a smaller delay in onset date. For example, in the case of 2 [1] dekad delay in onset date, D for AFI risk level 5 is 0.43 [0.32], whereas for AFI risk level 4, it is 0.26 [0.16], with the values being lowest in the case of AFI risk level 1, 0.03[0.02].

#### 3.4.2 Multivariate regression between NDVI standardized anomaly and onset date anomaly

In addition to examining the effect of onset date anomaly on the probability distribution function of NDVI^Z^, which shows that delay in onset can substantially increase the probability of NDVI^Z^ being below -1, we also performed a multivariate regression between onset dates, anomaly, and NDVI^Z^. This analysis further illustrates the influence of delay in onset dates on NDVI^Z^.

As described in section 2.4, we conducted this analysis with (i) onset date anomaly calculated relative to median onset date (“*Regression-1*”, Fig 6), as well as (ii) by treating onset date anomaly as a binary discrete variable (“*Regression-2*”, Fig 6), where onset date is treated as a binary variable of value 0 (on time or early onset) or 1 (more than 2 dekad delay in onset date). Estimates obtained from “*Regression-1*” quantify linear relationships between both variables, and estimates obtained from “*Regression-2*” use the binary variable to approximate non-linear relationships.

We fit models on data including all the AU2 in SSA, and we also fit separate models for each region/season (Fig 6). Estimated coefficients on the continuous and binary onset date values along with 95% confidence intervals are shown in Fig 6.

For the models fit on all regions (All regions), the estimated coefficient on *β*_1_ from “*Regression-1*” (red lines in Fig 6) is negative (*β*_1_ = -0.067), indicating that each dekad of delay in onset date will, on average, reduce end of season NDVI^Z^. When each of the regions/seasons are examined separately, the relationship between onset date anomaly and NDVI is strongest in the case of EA, especially for “short rains” season (*β*_1_ = -0.277), which supports the results from the analysis presented in the previous sections. For WA and SA, the estimated coefficients are smaller than those in other regions and the 95% confidence intervals suggest that the coefficients for WA and SA are not statistically significant.

Estimates of *β*_2_ from “*Regression-2*” (Fig 6), which uses a binary indicator of “late” season starts to approximate potential non-linear relationships, are generally larger than those from “*Regression-1*.*”* For example, in the model fit on EA “short rains,” onset date that is late by more than two dekads will, on average, lower end of season NDVI by one standard deviation (*β*_2_ = -1.23). This difference in magnitude between the coefficients in the model that uses a continuous measure (*β*_1_) and the model that uses a binary measure (*β*_2_) indicates that there may be a key threshold effect at which delay in onset date begins to impact NDVI scores.

Finally, we also calculated the estimates from “*Regression-1*” for each of the AU2 individually (S4 Fig) and, in general, we see a strong and spatially coherent, negative impact of onset date delays on NDVI anomalies in EA.

## 4 Concluding remarks

Food-insecure households across semi-arid monsoonal areas of SSA typically experience a seasonal cycle in their food-security conditions, which rapidly declines at the onset of drought. The early identification of drought is needed to support appropriate action to mitigate the adverse impacts of AFI on lives and livelihoods. Optimal early warning systems rely on a progression of alarms that begin several months before the start of the rainy season and end after the conclusion of the rainy season. The onset date offers a first glimpse into a rainy season performance using observational rainfall. By construct, it occurs several months before the mid-season, pre-harvest lean period, and up to a year before the next lean season when AFI risks are typically higher. Given the importance of the onset date, this study investigates how onset date is linked to seasonal drought conditions in SSA.

For the purposes of this study, peak NDVI following a rainy season is used as a proxy for drought conditions, particularly for the purposes of crop and pastoral conditions. While NDVI is frequently used as an indicator of, or to, model crop production and pastoral conditions in both research and operational settings [18, 45, 46, 50], there is not always a direct relationship between NDVI and food production. However, given the prevalence of NDVI as a monitoring tool, we assert that identifying the relationship between onset and peak NDVI is valuable to food security analysts who help provide food-insecurity outlook assessments (such as those provided by FEWS NET) to inform decisions that mitigate adverse impacts of food insecurity.

This study demonstrates that, specifically in the regions of SSA, which in the last decade have most often needed emergency food assistance, onset date can be a reliable early indicator of seasonal drought conditions. The results show that, in general, (i) negative median correlation exists between onset date and peak NDVI, especially in the case of AU2 with AFI risk level 5 (i.e., most food insecure AU2) of EA region, where it ranges between -0.42 to -0.68; (ii) as the risks of AFI decreases, this relationship becomes weaker; (iii) peak NDVI tends to be smaller in the case of latest onset date versus earliest onset date, with the difference in peak NDVI reaching ~-1.5 standardized anomaly in case of EA; (iv) delay in onset date by more than 1 dekad makes the drought conditions (in terms of NDVI^Z^) more likely, especially in the case of the AU2 with AFI risk level 5, where the chances of NDVI^Z^ being below <-1 increases by more than 2 fold if the onset date is delayed by more than 1 dekad and by more than 3 fold (to >50% probability) if it is delayed by more than 2 dekads. (v) regression analysis between NDVI^Z^ and onset date anomalies indicates a negative influence of onset date anomalies on NDVI^Z^, which is statistically when all the regions/seasons are binned together and especially strong (and statistically significant) in the case of the EA region. Results identified an especially strong relationship between rainfall onset and peak NDVI in the EA “short rains” season. This has useful implications for drought early warning during that season, as there are also well-established long-lead time rainfall prediction opportunities associated with the El Niño-Southern Oscillation and the Indian Ocean Dipole [62].

In the time that it takes a season to fail, millions of dollars in livestock can be lost, and millions of people may be exposed to the ravages of AFI. This study reveals onset date as an important early indicator of drought, particularly in the regions of SSA, which are at the highest risk of AFI, and hence, this study has direct implications in terms of supporting efforts to timely mitigate the most adverse impacts of AFI. For example, based on these results, and in particular, for the region of EA both for the “long rains” and “short rains” season, onset date can be closely watched starting a few dekads before the climatological start of the season to track early arrival or lateness of the rainy season. This information could be used to augment existing efforts to use remote sensing-based products to provide early season yield estimates [63, 64]. In the case of delay in the onset date, maps depicting probabilistic estimates of seasonal drought conditions can be provided to trigger enhanced monitoring, verification with field reports, and early planning for relief efforts. Estimates of drought conditions can be provided using past analog years with similar levels of delay in onset date and/or driving models, such as WRSI, with anticipated delay in onset date to get an estimate of crop water stress at the end of the season [16, 31]. Moreover, the lead time for this early warning can be increased by combining observed rainfall with rainfall forecasts at 2 weeks (weather forecasts) [32] or monthly scale [33] (subseasonal scale) to get an even earlier outlook for possible delay in season than what is possible based solely on observations. Additionally, the methods and data sets used in this study are applicable and available globally, which can allow for the investigation of the value of onset date as an early warning tool for other food-insecure regions across the globe. Finally, future work could build on our results by examining the relationship between onset date and high-resolution crop yield data, or other dynamic indicators of food insecurity, such as mid-season grain or livestock prices.

## Supporting information

S1 Fig Climatological month of onset (left panel) and peak NDVI month for the administrative units-2 in East, West, and Southern Africa regions that are monitored by FEWS NET. (TIFF)

S2 Fig Same as the scatter plots in Fig 3 but the X axis is onset date anomaly is in the units of dekads. For visual clarity the X axis is restricted to be between -6 to +6 dekads (i.e. two monthly early onset to two months delayed onset).
(TIFF)

S3 Fig Variability of correlation between onset date and seasonal precipitation, as well as between seasonal precipitation and peak NDVI in the following months for East, West, and Southern Africa, binned based on AFI risk levels. (TIFF)

S4 Fig Estimates from “Regression-1”, between NDVI^Z^ and onset date anomaly, for individual AU2s for all regions/seasons. (TIFF)
