# Supplementary figures and images for: A slow rainy season onset is a reliable harbinger of drought in most food insecure regions in Sub-Saharan Africa
Source: PLoS One. 2021 Jan 20;16(1):e0242883. doi: 10.1371/journal.pone.0242883 (PMC7816988; doi:10.1371/journal.pone.0242883)

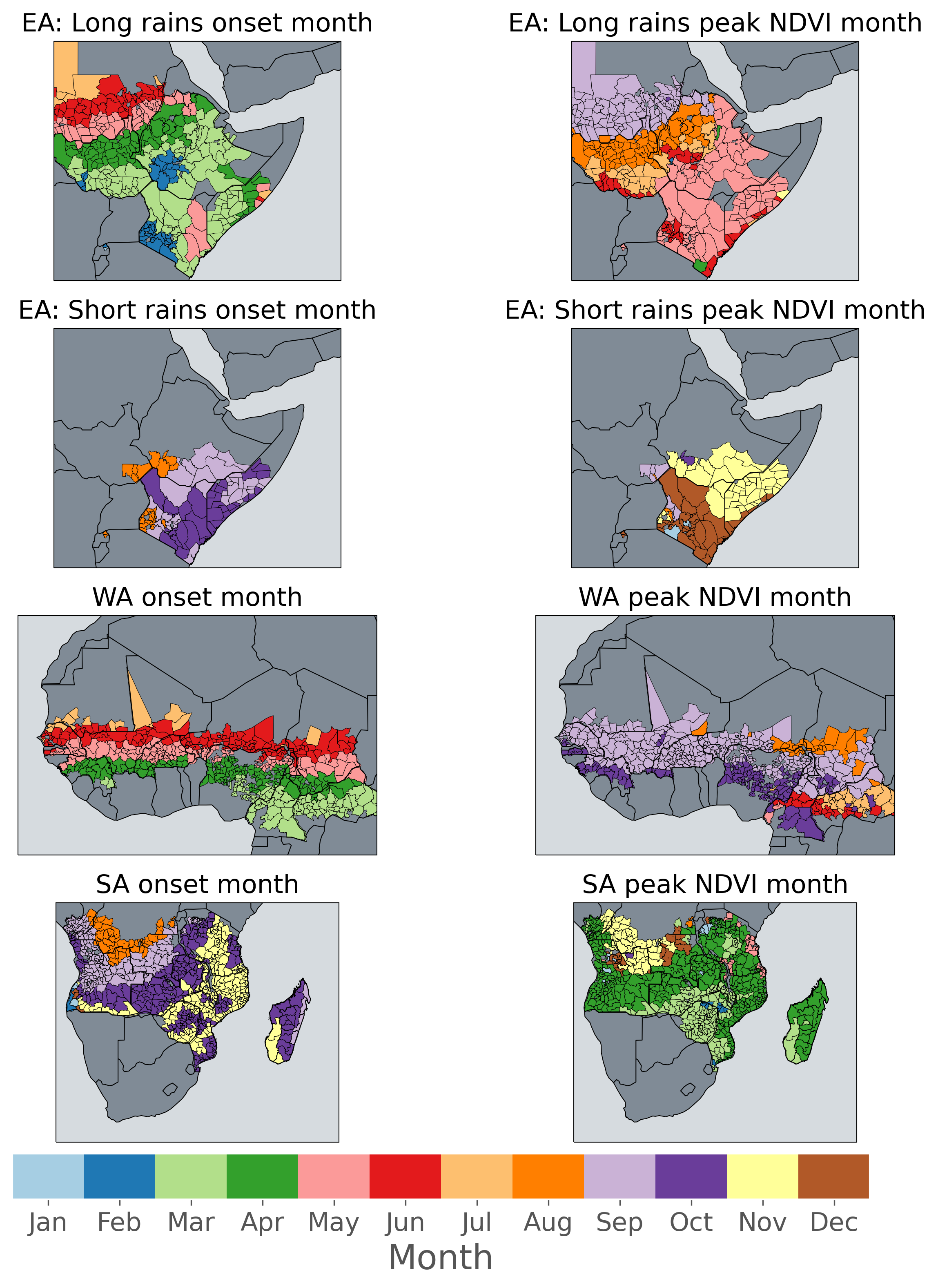

Supplement: S1 Fig — (TIFF) [file pone.0242883.s001.tiff]

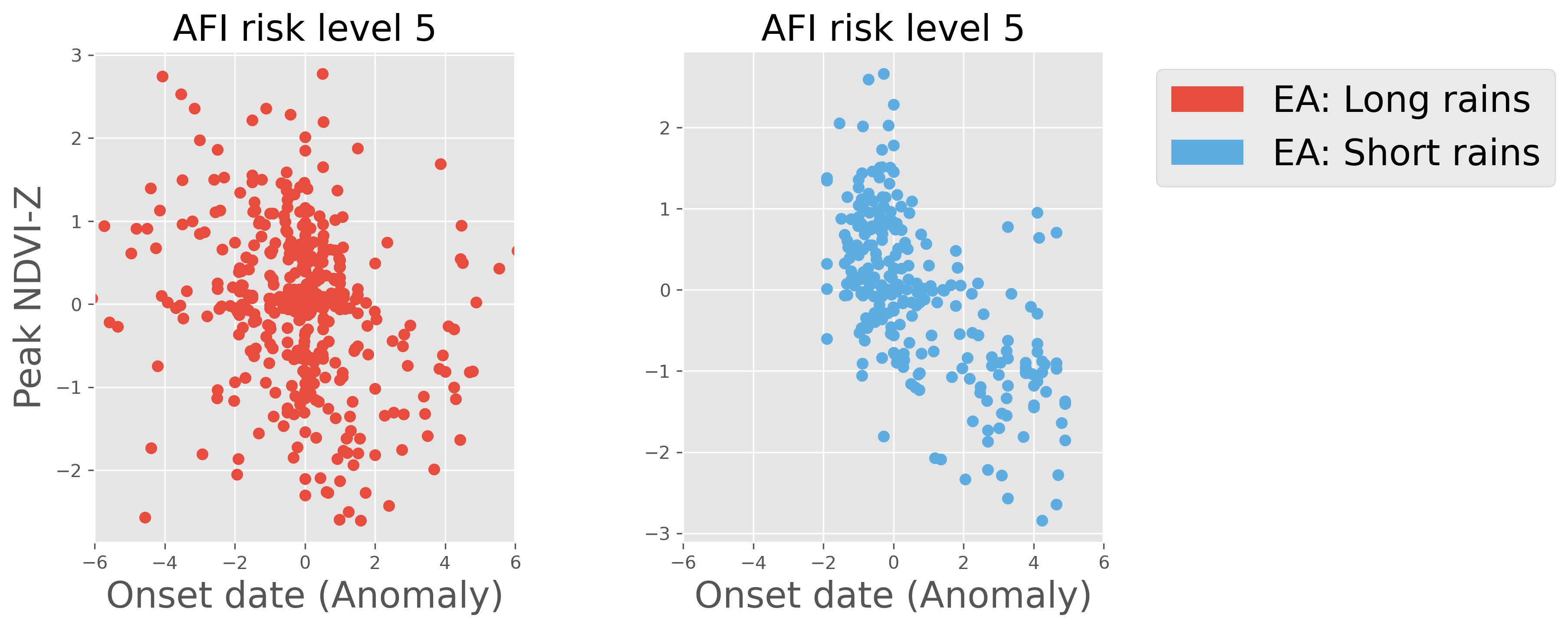

Supplement: S2 Fig — For visual clarity the X axis is restricted to be between -6 to +6 dekads (i.e. two monthly early onset to two months delayed onset). (TIFF) [file pone.0242883.s002.tiff]

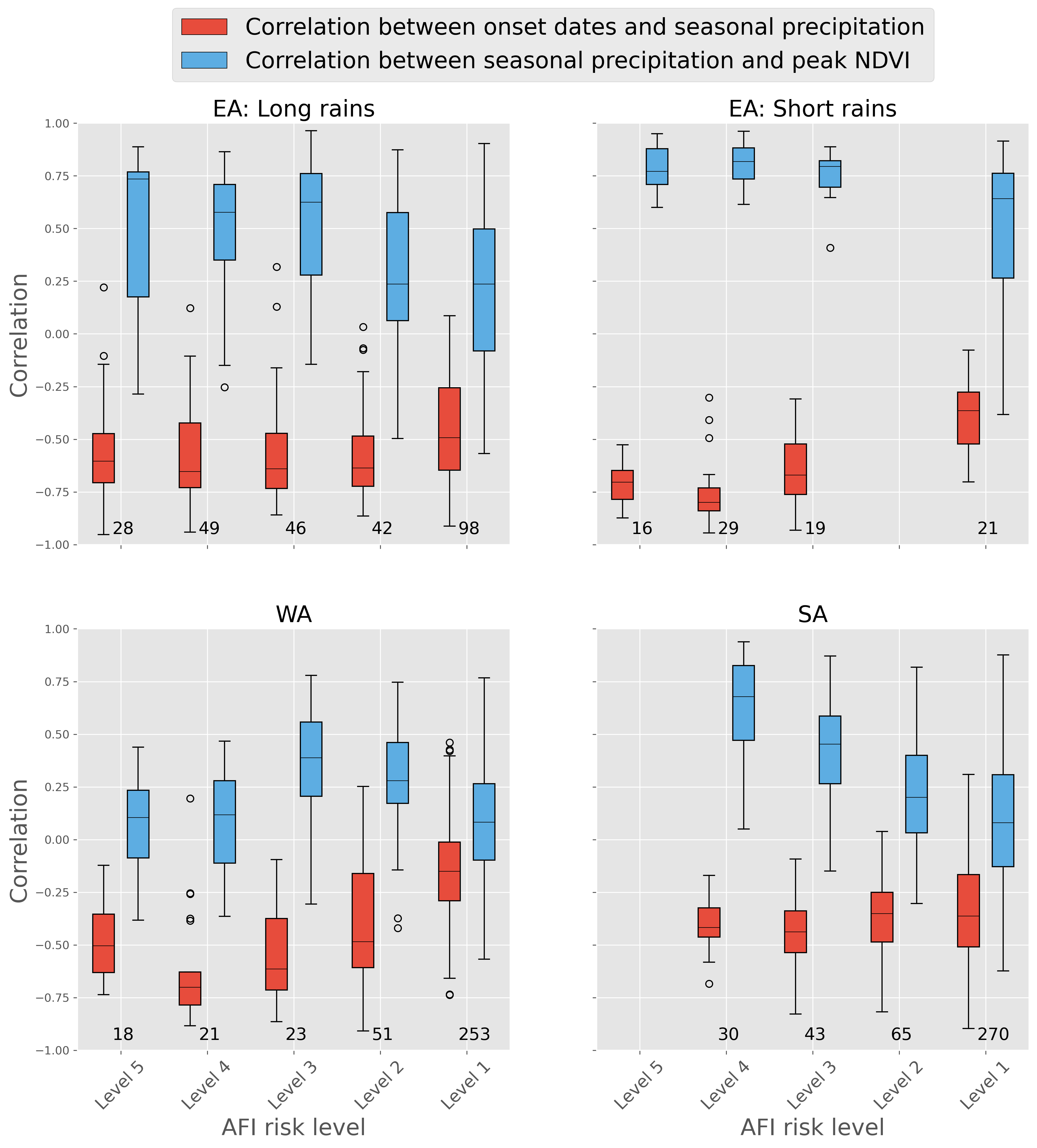

Supplement: S3 Fig — (TIFF) [file pone.0242883.s003.tiff]

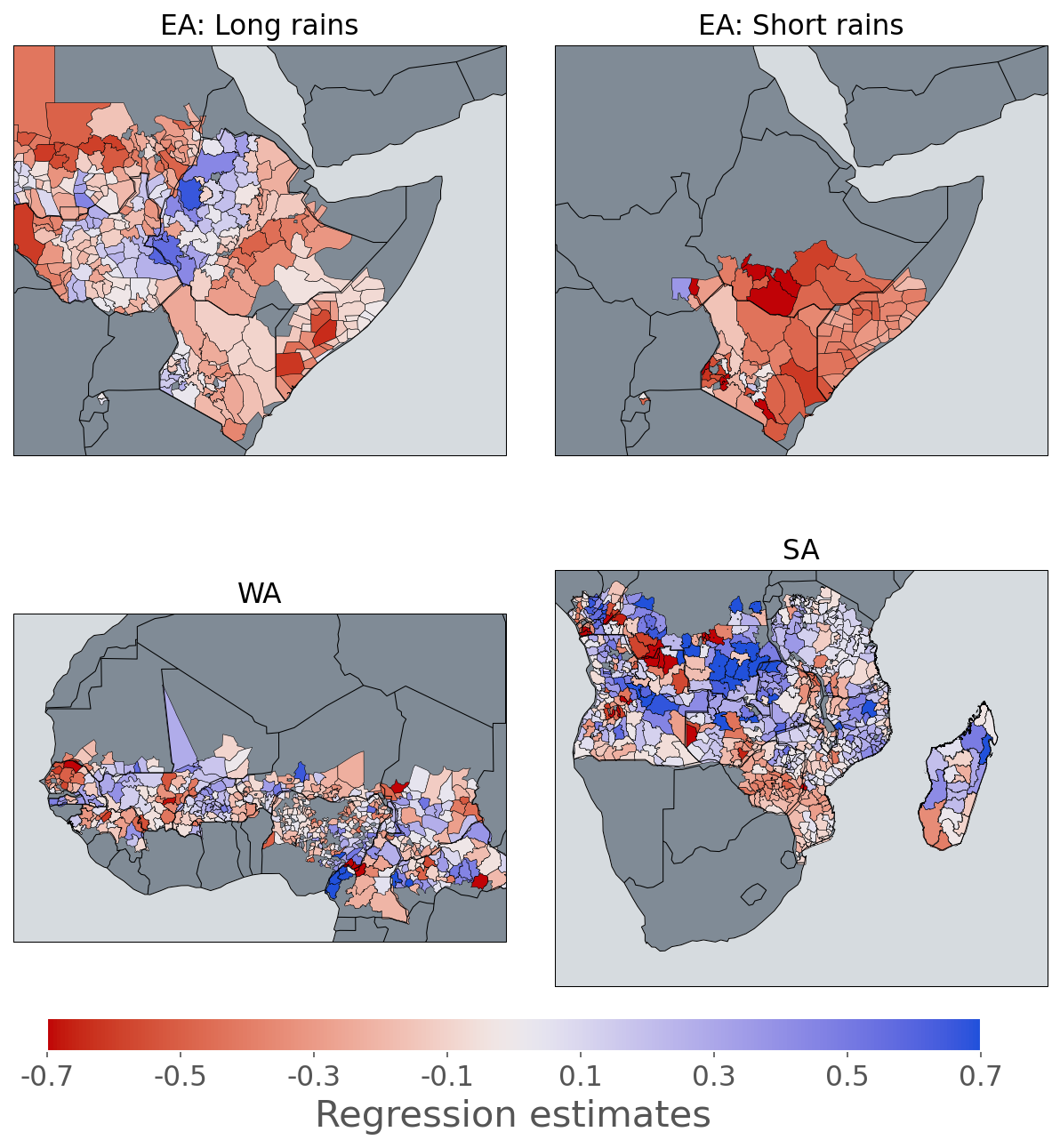

Supplement: S4 Fig — (TIFF) [file pone.0242883.s004.tiff]
